# Forecasting individual risk for long-term Posttraumatic Stress Disorder in emergency medical settings using biomedical data: A machine learning multicenter cohort study

**DOI:** 10.1016/j.ynstr.2021.100297

**Published:** 2021-01-18

**Authors:** Katharina Schultebraucks, Marit Sijbrandij, Isaac Galatzer-Levy, Joanne Mouthaan, Miranda Olff, Mirjam van Zuiden

**Affiliations:** a Vagelos School of Physicians and Surgeons, Department of Emergency Medicine, Columbia University Medical Center, New York, NY, United States of America; Data Science Institute, Columbia University, New York, New York, USA; b Vrije Universiteit, Department of Clinical, Neuro- and Developmental Psychology; Amsterdam Public Health Research Institute, World Health Organization Collaborating Centre for Research and Dissemination of Psychological Interventions, Amsterdam, the Netherlands; c Department of Psychiatry, New York University School of Medicine, New York, New York, USA; d Department of Clinical Psychology, Institute of Psychology, Faculty of Social and Behavioural Sciences, Leiden University, Leiden, the Netherlands; e ARQ National Psychotrauma Centre, Diemen, the Netherlands; f Department of Psychiatry, Amsterdam University Medical Centers, Location Amsterdam Medical Center, University of Amsterdam, Amsterdam Public Health Research Institute and Amsterdam Neuroscience Research Institute, Amsterdam, the Netherlands

**Keywords:** PTSD, Traumatic stress, Biomarkers, Prognosis, Machine learning, HPA axis, Pharmacotherapy, Thyroid hormones

## Abstract

The necessary requirement of a traumatic event preceding the development of Posttraumatic Stress Disorder, theoretically allows for administering preventive and early interventions in the early aftermath of such events. Machine learning models including biomedical data to forecast PTSD outcome after trauma are highly promising for detection of individuals most in need of such interventions. In the current study, machine learning was applied on biomedical data collected within 48 h post-trauma to forecast individual risk for long-term PTSD, using a multinominal approach including the full spectrum of common PTSD symptom courses within one prognostic model for the first time. N = 417 patients (37.2% females; mean age 46.09 ± 15.88) admitted with (suspected) serious injury to two urban Academic Level-1 Trauma Centers were included. Routinely collected biomedical information (endocrine measures, vital signs, pharmacotherapy, demographics, injury and trauma characteristics) upon ED admission and subsequent 48 h was used. Cross-validated multi-nominal classification of longitudinal self-reported symptom severity (IES-R) over 12 months and bimodal classification of clinician-rated PTSD diagnosis (CAPS-IV) at 12 months post-trauma was performed using extreme Gradient Boosting and evaluated on hold-out sets. SHapley Additive exPlanations (SHAP) values were used to explain the derived models in human-interpretable form.

Good prediction of longitudinal PTSD symptom trajectories (multiclass AUC = 0.89) and clinician-rated PTSD at 12 months (AUC = 0.89) was achieved. Most relevant prognostic variables to forecast both multinominal and dichotomous PTSD outcomes included acute endocrine and psychophysiological measures and hospital-prescribed pharmacotherapy.

Thus, individual risk for long-term PTSD was accurately forecasted from biomedical information routinely collected within 48 h post-trauma. These results facilitate future targeted preventive interventions by enabling future early risk detection and provide further insights into the complex etiology of PTSD.

## Highlights

• Accurate PTSD prognosis early post-trauma is required for effective preventive interventions.
• We performed prognostic modeling of PTSD risk in emergency department patients.
• Machine learning was applied on early biomedical data to forecast PTSD at 12 months.
• Good prediction of multinominal PTSD course and PTSD diagnostic status was achieved.
• Endocrine and physiological measures and pharmacotherapy were relevant features.

## 1 Introduction

Posttraumatic Stress Disorder (PTSD) is a common consequence of traumatic events , characterized by involuntary trauma re-experiencing, avoiding trauma-related reminders, negative alterations in mood and cognitions, hyperreactivity and arousal (Shalev et al., 2017). Next to impaired daily functioning and quality of life, PTSD is associated with physical morbidity and mortality (Bisson et al., 2015). Theoretically, the first weeks post-trauma provide a unique window of opportunity for preventive or early interventions for PTSD (Bisson et al., 2019). Accumulating evidence shows such interventions are only beneficial if targeted to individuals at high risk for long-term PTSD (Shalev and Barbano, 2019). Yet, currently no accurate methods available for individual risk classification of long-term PTSD outcome immediately after trauma have been implemented in routine clinical care. This not only hampers accurate targeting of the currently available evidence-based early intervention towards those most in need, but also hinders reliable investigation of the efficacy of novel intervention strategies (Garcia and Delahanty, 2017).

Recent years have seen an increased interest into investigations of distinguishable common trajectories or courses of PTSD symptoms across a designated follow-up period, instead of investigating diagnostic PTSD status at the end-point of a follow-up period only. A systematic review and meta-analysis of these individual studies concluded that resilient, recovering, chronic and delayed onset trajectories are commonly observed across trauma-exposed populations, with a clear imbalance in the percentage of trauma-exposed individuals following a resilient course versus a chronic, recovering or delayed course (Galatzer-Levy et al., 2018b). Among individuals with acute high PTSD symptoms, approximately 27% maintain non-remitting symptoms, while the majority recovers (Galatzer-Levy et al., 2018b). Vice versa, approximately 8% of individuals with initial low symptoms develop high symptoms with delayed onset (Galatzer-Levy et al., 2018b). Thus, based on early PTSD symptom severity alone, it is not possible to distinguish between non-remitting and recovering trajectories, nor between resilient and delayed trajectories (van de Schoot et al., 2018). To achieve effective PTSD prevention, it is pivotal to derive prognostic models that adequately capture this full spectrum of heterogenic common PTSD courses.

A growing number of prospective studies supports that individual differences in the functioning of biological systems associated with stress and threat perception, reactivity and recovery underlie differential vulnerability for PTSD (Heim et al., 2018; Hinrichs et al., 2019; Mellon et al., 2018; Michopoulos et al., 2019; Morris et al., 2016; Ressler, 2018; Schultebraucks et al., 2019). Most studies focused on markers reflecting current activity of the sympathetic nervous systems (SNS) (Morris et al., 2016) and hypothalamic-pituitary-adrenal axis (Van Zuiden et al., 2012). The predictive value of indices capturing the myriad of biological systems that bidirectionally interact with these primary stress response axes during and acutely after (traumatic) stress remain less often investigated. Within these, pre- and early post-trauma markers of immune activation and in vitro glucocorticoid receptor functioning within immune cells have been the most commonly investigated (Michopoulos et al., 2019; Van Zuiden et al., 2012). Additionally, early pharmacotherapy to treat injuries (e.g. opioids for pain, glucocorticoids for inflammation) or immediate psychological symptoms (e.g. benzodiazepines for acute anxiety or sleep disturbances) have been found to impact subsequent PTSD development (Amos et al., 2014; Frijling et al., 2019; Guina et al., 2015; Mouthaan et al., 2015; Sijbrandij et al., 2015).

However, thus far these findings have not been translated into applicable prognostic models and in general have had limited clinical impact yet. This is likely because the used data-analytic strategies within these prospective studies only permitted linear(-additive) inferences at the group-level between predictor variables and PTSD outcome. Therefore, these methods could not delineate the complex multivariate interactions between and within biological pathways expected to underlie PTSD symptom development. This methodological limitation can be overcome using machine learning (ML), which uses data-driven modeling to identify computational algorithms that recognize patterns and associations in complex interrelated data. To date, several studies support the promise of ML for early PTSD prognosis (Galatzer-Levy et al., 2014, 2017; Karstoft et al., 2015; Papini et al., 2018; Schultebraucks et al., 2020a; Wshah et al., 2019). These studies also showed that biomedical data, including endocrine and physiological markers and received pharmacotherapy, can provide high probabilistic information in these models. A recent US study developed an algorithm using routinely collected biomarkers from electronic medical records plus four questions about the psychological stress response that accurately discriminated heterogenous trajectories of PTSD in two independent clinical samples (Schultebraucks et al., 2020a).

However, these first studies did not forecast the full spectrum of common PTSD symptom courses following recent trauma exposure within one prognostic model (Schultebraucks and Galatzer-Levy, 2019). Different etiological mechanisms likely underlie non-remitting vs delayed trajectories, as well as recovery vs resilient trajectories upon trauma exposure (Galatzer-Levy et al., 2018a). Thus, it is to be expected that early risk classification for PTSD could be further improved by forecasting the whole course spectrum simultaneously, and such an approach may also shed further light on the mechanisms involved in PTSD pathogenesis. Therefore, in this multicenter cohort study in emergency department (ED) patients with (suspected) serious injury, we aimed to derive multinominal ML models for individual prognosis of the full spectrum of PTSD symptom courses over 12 months following trauma based on biomedical data collected upon hospital admission.

Building upon previous findings (Heim et al., 2018; Ressler, 2018), we adopted a hypothesis-driven approach for biomedical feature selection. First of all, we included information on physiological and endocrine markers reflecting SNS and HPA axis activation upon ED admittance. Secondly, based on previous findings within our own and other cohorts investigating group-level predictors we also included several categories of prescribed psychopharmacology (Mouthaan et al., 2015) within 48 h after admission. Based on cross-sectional literature in PTSD patients (Olff et al., 2006), we additionally included measures of hypothalamic-pituitary-thyroid (HPT)-axis activation and DHEAS levels upon ED admittance and additional categories for received pharmacotherapy.

Thirdly, seeing the ML approach allows for investigating of multivariate interactions, we further opted to include demographic, traumatic event and injury characteristics, and information on trauma history. These domains have been found to both predict subsequent PTSD course and diagnostic status by themselves, but importantly also have been shown to moderate associations between the biological markers under investigation and subsequent PTSD course (Galatzer-Levy et al., 2017; Walsh et al., 2013).

In contrast to previous research on predicting PTSD in ED patients (Schultebraucks and Galatzer-Levy, 2019; Schultebraucks et al., 2020a), we included only biomedical data with the potential to be routinely collected in the ED, as this would enable time-efficient and inexpensive individual risk classification early post-trauma, without the necessity of extensive psychological or biomedical assessment prior to classification. Moreover, we used the Clinician Administered PTSD Scale (CAPS-IV) (Weathers et al., 2004) to verify the predictions, which is the gold standard approach for prognostic modelling of PTSD, but often replaced by self-reports. In addition to the multinominal model based on self-reported symptoms, we also forecasted end-point PTSD diagnostic status (CAPS-IV cut-point diagnosis), to compare results between the resulting multinominal prognostic model and a binary decision on diagnostic status that is the most common approach in clinical practice.

Furthermore, to obtain potential correlates of PTSD pathogenesis we subsequently applied explainable ML to interpret the derived models by examining the complex associations between the biomedical variables deemed most relevant in the derived prognostic model and PTSD outcome.

## 2 Materials and methods

### 2.1 Participants

The data were collected in the framework of a larger study combining a prospective longitudinal cohort study on incidence and prediction of trauma-related psychopathology with an embedded randomized controlled trial aimed at preventing PTSD with a brief self-guided internet intervention based on cognitive behavioral therapy techniques in a subset of participants (“The Incidence, Prediction and Prevention of Post-trauma Psychopathology Study” (Trauma TIPS), ISRCTN registration number: 57754429) (Mouthaan et al., 2013, 2014a, 2014b, 2015).

The cohort consists of a consecutive sample from two level-1 Trauma Center sites (Academic Medical Center and Vrije Universiteit Medical Center) in Amsterdam, the Netherlands between September 2005 and March 2009. As of September 2007, participants were additionally asked to participate in the embedded RCT, but could also opt to participate in the cohort study only. Results detailing that end-point clinician-rated and self-reported PTSD symptom severity did not differ between intervention and control conditions are reported elsewhere, see Mouthaan et al. (2013).

Adults transported to the Trauma Centers by ambulance or helicopter with suspected severe injuries requiring specialized acute medical care were eligible for inclusion if they had experienced a potential traumatic event (DSM-IV PTSD criterion A1) and had Dutch language proficiency. Exclusion criteria were: current severe psychiatric symptoms (psychosis or schizophrenia; severe personality disorders; or injuries resulting from deliberate self-harm); current severe neurological disorder and/or moderate-severe Traumatic Brain Injury (Glascow Coma Scale (GCS) score <13 (Teasdale and Jennett, 1974) (potentially influencing participants’ reporting reliability)); permanent residency outside the Netherlands. The study was approved by institutional review boards of both centers. Eligibility was assessed for N = 1729 patients, of whom N = 256 (14.8%) met exclusion criteria. N = 852 provided informed consent and were included in the larger study (49.3%).

For the current study, we included N = 417 (N = 238 cohort study only, N = 95 RCT intervention condition, N = 84 RCT control condition) with ≥2 valid self-reports on PTSD symptoms across follow-up assessments to allow for reliable estimation of the course of PTSD symptom severity. Upon inclusion, participants were on average 46.09 ± 15.88 years old and 62.8% were males (N = 262). Most common event types were road traffic accidents (62.4%); falls from height (16.1%); work-related accidents (12.0%) and physical assault (4.2%).

### 2.2 Procedures

Upon arrival at the Trauma Unit and initial medical examination, hospital staff collected blood samples for stress hormone assessment. Hospital registrations were used to select potentially eligible patients. Patients meeting exclusion criteria based on the patient records or information from treating medical staff in case of ongoing hospitalization were not invited to participate. Further screening for eligibility and recruitment was performed in hospital in case of ongoing hospitalization or otherwise via telephone within 72 h post-injury (T0). All further assessments consisted of face-to-face interviews and self-report questionnaires. All participants were invited for follow-up assessments, irrespective of RCT participation, and had access to care as usual.

At a face-to-face baseline assessment (T1), preferably scheduled at approximately one-week post-injury, participants gave written and oral informed consent. If severe psychiatric symptoms meeting the exclusion criteria were disclosed during these assessments, participation was discontinued. Potential intervention initiation in case of RCT-randomization to the intervention condition took place after this assessment. Follow-up assessments were preferably scheduled at 1 months (T2), 3 months (T3, initiated after study commencement, therefore subset only), 6 months (T4) and 12 months (T5) post-injury (Supplementary Fig. 1). However, due to practical issues related to the oftentimes severe injuries in the sample, T1 and T2 assessments frequently occurred later than preferred.

For the current study, T1 and T2 data collected ≥32 days and ≥62 days post-injury respectively were excluded. For follow-up assessments T3-T5, data collected at inconsistent time intervals from the rest of the sample (i.e. more than two standard deviations from the mean) were excluded (as proposed by Galatzer-Levy et al. (2013)). Sample size and timing relative to the injury for each assessment were: T1: N = 340, 21.61 (16.75) days; T2: N = 374, 46.43 (17.72) days; T3: N = 214, 107.87 (22.04) days; T4: N = 305, 213.35 (35.63) days; T5: N = 288, 422.69 (62.94) days. At T5, CAPS-IV interview data were available for N = 273 participants, with N = 12 (4.4%) of interviewed participants fulfilling diagnostic criteria for current PTSD. We did not observe a difference in end-point PTSD status between RCT participants (3.9%) and non-participants (4.7%, X2(1) = 0.086, p = 1.000), nor between the intervention (3.7%) and control condition (4.2%) amongst RCT participants (X2(1) = 0.014, p = 1.000).

### 2.3 Measures

#### 2.3.1 Outcome

End-point PTSD diagnostic status was determined using the CAPS-IV (Weathers et al., 2004). Total symptom severity was calculated by summing frequency and intensity scores for all 17 items, each representing one DSM-IV PTSD symptom. We followed DSM-IV criteria regarding minimum number of required symptoms (frequency≥1, intensity≥2), and additionally required a total symptom severity score of ≥45 (Weathers et al., 2004).

Self-reported PTSD symptom severity in the past week was measured at each assessment, using the 22-item Impact of Event Scale-Revised (IES-R) (Weiss and Marmar, 1997). Total symptom severity was determined by summing all item scores.

#### 2.3.2 Prognostic variables

We included 51 variables capturing routinely collectable information within the first 48 h after ED admission on several domains. We included 2 demographic variables (age, gender), 2 variables summarizing trauma history (total number and impact of prior traumatic experiences) and 8 variables concerning the current traumatic event (type of trauma (categorical); perceived life threat during the incident (binary); 6 binary variables concerning whether others were injured or deceased during the incident, whether participants were directly confronted with and whether they knew the injured or deceased individuals). Furthermore, we included 10 injury characteristics (any injury sustained (binary); any head injury sustained (binary); physician-rated Injury Severity Score (ISS) total score and severity category (Baker et al., 1974)); Glasgow Coma Scale total score and 3 subscale scores (Teasdale and Jennett, 1974); intubation status (binary); self-reported amnesia (binary)) and 3 medical treatment characteristics (24-h clock time at ED admittance; Intensive Care Unit admission status after ED discharge (binary); admission status elsewhere in hospital after ED discharge (binary)).

We also included 3 psychophysiological (vital) measures (heart rate; systolic blood pressure; respiration rate) and 5 endocrine measures (cortisol; dehydroepiandrosterone sulfate (DHEAS); thyroid-stimulating hormone (TSH); free triiodothyronine (FT3)*;* and free thyroxine (FT4)) (Mouthaan et al., 2014a, 2015), collected immediately upon ED admittance. Finally, we included 18 variables concerning received pharmacotherapy, representing the number of doses hospital-prescribed within the first 48 h post-admittance within the following categories: opiate analgesics; opiate anesthetics; non-opiate analgesics; non-opiate anesthetics; systemic glucocorticoids; ACE inhibitors; beta-adrenergic receptor blockers; vasoconstrictors; vasodilators; anticoagulators; antihistamines; antibiotics; cholesterol inhibitors; gabapentin; other anticonvulsants; benzodiazepines; other hypnotics; other psychotropic medication (Mouthaan et al., 2015).

### 2.4 Statistical analyses

#### 2.4.1 Latent Growth Mixture modeling (LGMM)

We used LGMM to identify distinct PTSD symptom trajectories. A series of unconditional LGMMs were applied to determine how many distinct latent classes best describe IES-R PTSD symptom severity trajectories over 12 months post-trauma. Individuals were assigned to one of the identified trajectories based on most likely class membership (highest posterior probability). To identify the best fitting unconditional LGMM, a nested model approach was applied in which a progressive number of classes were analyzed until adding additional classes did not further improve model fit. Trajectory shape was investigated by examining freely estimated intercept, linear and quadratic slopes. Model fit was evaluated by assessing sample-size adjusted Bayesian Information Criterion, Akaike Information Criterion indices, and Vuong-Lo-Mendell-Rubin Likelihood. Entropy was used to assess clarity of class specification. Besides model fit, parsimony and interpretability were used to select the optimal number of classes. Analyses were performed using Mplus version 7 (van de Schoot et al., 2017). Result reporting is based on the GROLTS-checklist (van de Schoot et al., 2017) for LGMM.

#### 2.4.2 Prognostic ML models

First categorical variables were dummy coded, continuous variables were normalized to range 0–1, and variables with near-zero variance were considered empty information and removed. We applied the same approach for the models predicting PTSD symptom trajectory and end-point PTSD diagnostic status. The sample was randomly split into 80% training set and a 20% hold-out set for internal model validation on data unseen by the model during the model building process, stratified for the outcome variable to achieve a distribution in training and hold-out sets matching the overall distribution. Missing values were imputed using bagged imputation during 5 times 3-fold cross-validation for hyperparameter tuning to prevent information leakage from training to hold-out set (Kuhn and Johnson, 2013). Preprocessing steps were separately applied to training and hold-out set. During model building on the training set, different resampling techniques (repeated cross-validation) were explored to guard the hyperparameter search against overfitting. eXtreme Gradient Boosting (XGBoost) (Chen and Guestrin, 2016) was used to build a multivariable predictive model. XGBoost is a tree-based ensemble method that is based on decision trees and applies gradient descent optimization to minimize training error. Model tuning and final model selection only used information from training sets, hold-out sets were strictly kept apart for internal model validation. Random oversampling of minority classes (Japkowicz, 2000) was explored as recommended (López et al., 2013). Model building and evaluation was performed with R package ‘caret’ (Kuhn and Johnson, 2013) in 3.6.0 using RStudio Version 1.2.1335. For model selection based on the training performance for the trajectory model, the average one-versus-all Area Under the Receiver Operating Characteristic Curve (AUC) from the ‘multiClassSummary’ function in the ‘caret’ package (Kuhn and Johnson, 2013) was used as metric. For model validation of the trajectory model on the hold-out set, the average one-versus-all AUC (multi-class AUC) was assessed based on Hand and Till (2001) via R package ‘pROC’ (Robin et al., 2011). For the end-point model we used AUC as metric (Kuhn and Johnson, 2013). We additionally calculated the cumulative gain, also known as accuracy ratio (Kuhn and Johnson, 2013). The cumulative gain is a robust metric well suited for imbalanced data, due to a low prevalence of the outcome-of-interest, and illustrates how much better the model is able to discriminate between outcomes compared to a random classifier. Result reporting is based on the CHARMS-checklist (Moons et al., 2014) for prognostic models.

#### 2.4.3 Explainable ML

To examine variable importance ranking and to interpret the decision rules incorporated in the model on PTSD diagnostic status at 12 months, we applied methods for explainable ML by using SHAP (SHapley Additive exPlanation) values for decision tree-based non-linear models (Lundberg and Lee, 2017). For each variable, it was analyzed how the model's prediction would have been without compared to with the variable. SHAP values per variable were averaged over every possible order of including the variable into the model while controlling for the influence of order.

#### 2.4.4 Secondary analyses

Secondary analyses were performed to exclude that the embedded RCT influenced our findings. First, we investigated whether effects of the RCT on LGMM results. Thereafter we performed sensitivity analyses, excluding participants in the RCT intervention group from the prognostic model for end-point PTSD.

## 3 Results

### 3.1 PTSD symptom trajectories

**Fig. 1:**
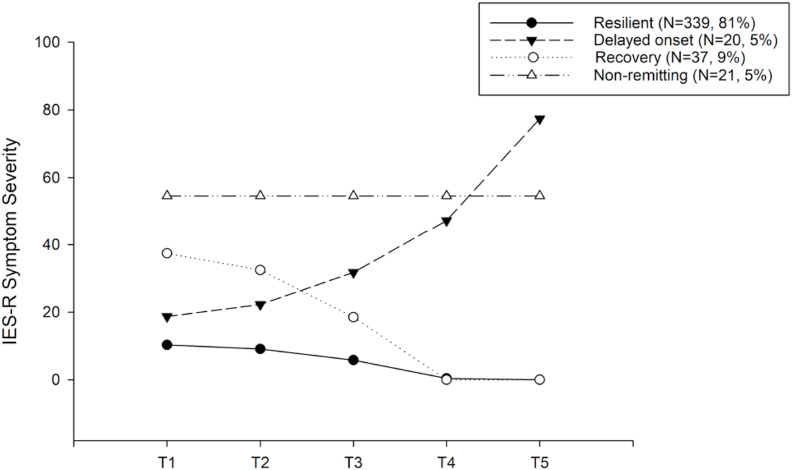
Unconditional model of latent trajectories based on self-reported PTSD symptoms (IES-R) through 12 months after Emergency Department admission. Presented are PTSD symptoms for each class at each respective assessment point represent estimated means from the Latent Growth Mixture model. Time points on the x-axis represent 46.43 (17.72) days; (T2); 107.87 (22.04) days (T3); 213.35 (35.63) days T4); 422.69 (62.94) days (T5) after Emergency Department admission.

The LGMM analyses showed that a four-class solution with quadratic term provided best model fit, indicating four distinct PTSD symptom trajectories over the course of 12 months post-trauma (Table S1). Qualitatively, the trajectories can be described as resilient (“continuous minimal symptoms”), recovery (“high initial symptoms but remitting”), delayed (“delayed increasing symptoms”), and non-remitting (“stable high symptoms”) (Fig. 1). Most participants were assigned to the resilient trajectory, whereas the other trajectories each comprised a minority of participants (Table 1). RCT participation and condition did not impact the results of the LGMM (see supplementary results). The two trajectories thought to related to adverse outcome, i.e., chronic and delayed, significantly differed from the resilient and recovery trajectories in terms of clinician-rated PTSD symptom severity and diagnostic status, as well as in self-reported Quality of Life, at the final assessment at 12 months (see Supplementary Table 2).

**Table 1:** Distribution of the observed outcome regarding self-reported PTSD symptom trajectory incidence in training set and hold-out set.

	Class 1: Resilient	Class 2: Delayed	Class 3: Recovery	Class 4: Chronic
Incidence in total sample (N = 417)	N = 339 (81%)	N = 20 (5%)	N = 37 (9%)	N = 21 (5%)
Incidence in training set (N = 335)	N = 272 (81%)	N = 16 (5%)	N = 30 (9%)	N = 17 (5%)
Incidence in hold-out set (N = 82)	N = 67 (82%)	N = 4 (5%)	N = 7 (8%)	N = 4 (5%)

### 3.2 Prognostic models

**Fig. 2:**
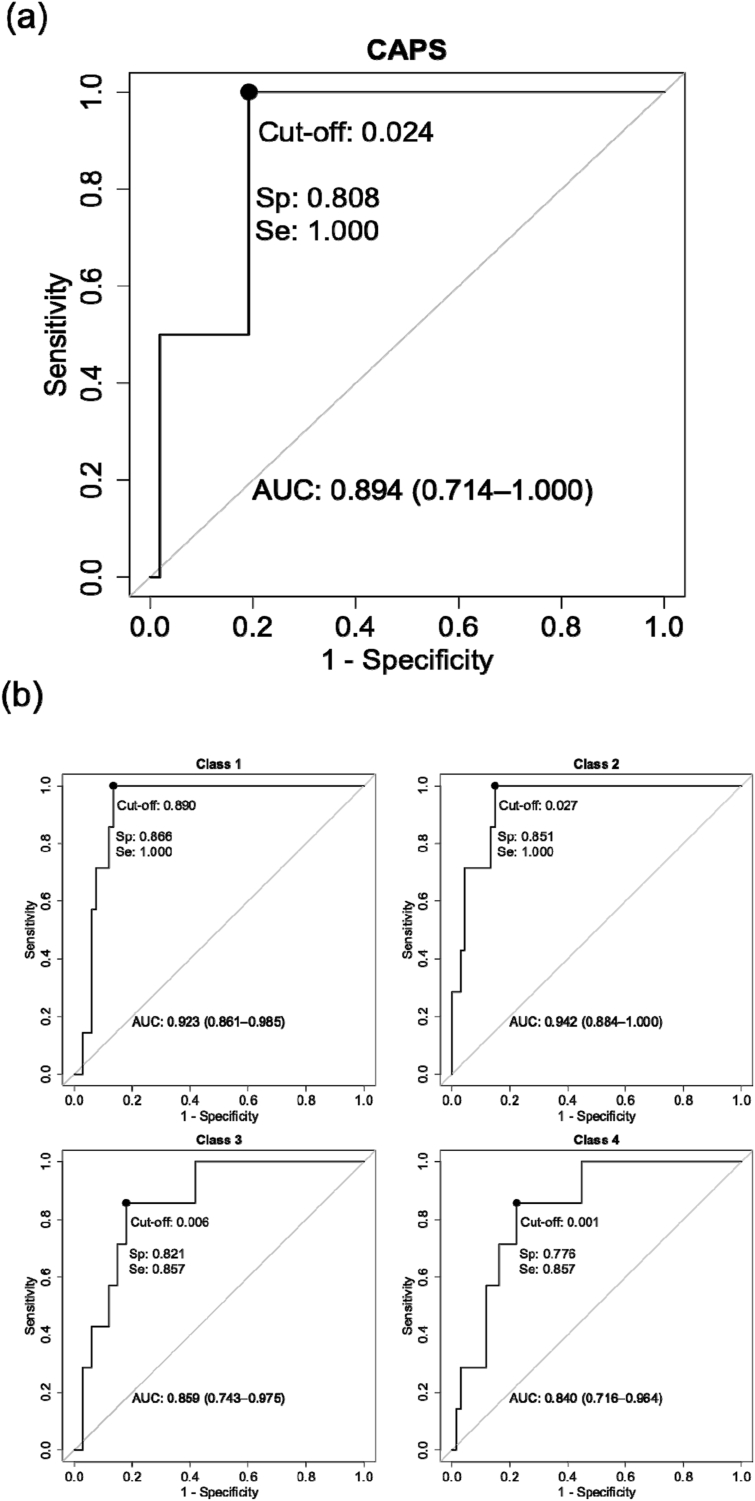
Receiver-Operating Characteristic Curves for the prediction of PTSD diagnostic status at 12 months after Emergency Department admission (Fig. 2a) and for the four PTSD symptom trajectories through 12 months after Emergency Department admission (Fig. 2b) in the hold-out set.

For the prognostic model predicting PTSD trajectory membership over 12 months-post trauma, the algorithm yielded a multiclass AUC of 0.89 in the hold-out set (multiclass accuracy = 0.79, 95% CI: 0.69–0.87; precision = 0.83; Fig. 2b). For the prognostic model predicting CAPS-IV PTSD diagnostic status at 12 months post-trauma an AUC of 0.89 (95% CI: 0.71–1) was obtained in the hold-out set (sensitivity = 1.00, 95%CI: 1-1; specificity = 0.81, 95% CI: 0.71–1; precision = 0.97, Fig. 2a). Cumulative gain is presented in Supplementary Figs. 2 and 3. In a sensitivity analysis, similar results for the end-point PTSD model were obtained when excluding the RCT intervention group (N = 95) from the analyses (AUC = 0.88, 95% CI: 0.75–1; sensitivity = 1.00, 95% CI: 1-1; specificity = 0.83, 95% CI: 0.71–0.98; precision = 0.96, Supplementary Fig. 7).

### 3.3 Model interpretation

**Fig. 3:**
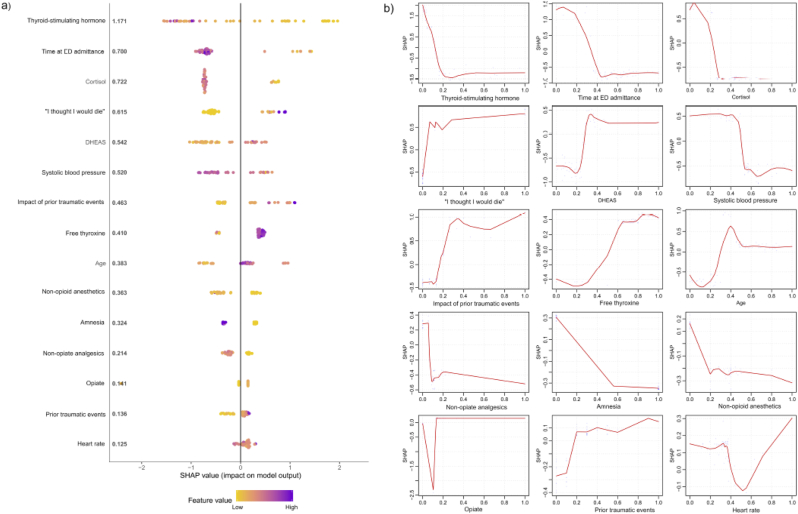
SHapley Additive exPlanations (SHAP) values for the model predicting PTSD diagnostic status at 12 months after Emergency Department admission in the hold-out set. Fig. 3A visualizes the SHAP values per feature for the 15 most important contributing variables within the model. At the Y-axis, the variables are ordered according to their importance to differentiate between PTSD and non-PTSD diagnosis with CAPS-IV at 12 months (mean absolute SHAP value per feature is depicted immediately after the feature name, larger SHAP values represent a higher importance to the model). The x-axis represents the probability for end-point PTSD diagnosis as log odds. Each colored point represents a point estimate of the impact of a predictor on the predicted value for a single participant. Fig. 3B displays the decision rule for each feature within the model, with the 0-value on the X-axis reflecting the lowest value of the feature and the 1-value representing the highest value of the feature respectively.

Fig. 3 depicts the 15 most important predictor variables for forecasting end-point PTSD diagnostic status, the directions of the associations between these variables and outcome classification and the applied non-linear classification decision rules (also see Fig. S4). Of these 15 variables, 10 (66.7%) were also among the 15 most important variables to differentiate between symptom trajectories (Supplementary Fig. 5). Tables S3 and S4 depict descriptive statistics for these most important variables for participants grouped by end-point PTSD diagnostic status and assigned trajectory respectively. Additionally, Fig. S6 shows the interaction effects between the most important variables contributing to the end-point PTSD model on the classification using a partial dependence plot (Friedman, 2001).

## 4 Discussion

Our results show that biomedical data collected immediately upon and within 48 h after ED admission can be used to forecast both PTSD symptom course over 12 months post-trauma and end-point PTSD diagnostic status with high predictive power. This finding that high prognostic accuracy can be obtained from early biomedical data concurs with and extends findings from previous ML studies (Schultebraucks and Galatzer-Levy, 2019; Schultebraucks et al., 2020a). Importantly, our study is the first to apply multinomial prognostic ML models for PTSD outcome, allowing us for the first time to forecast the full spectrum of heterogeneous PTSD trajectories upon trauma-exposure within one prognostic model.

Forecasting trajectories of PTSD symptom course, derived from repeated assessments over the whole follow-up period, conveys more detailed information on heterogeneity in PTSD course than single time-point assessments and end-point diagnostic status. Importantly, the four identified trajectories concur with meta-analytic findings on commonly observed PTSD symptom trajectories across populations from a variety of traumatic events (Galatzer-Levy et al., 2018b). As typically observed, especially the non-remitting and delayed trajectories are substantially smaller than the resilient trajectory. The imbalance between the classes of the outcome would be challenging for most traditional statistical models but to ignore the heterogeneity in the outcome variable would mean to also ignore the clinical reality of heterogeneous responses to traumatic events. Our applied multi-nominal classification ML approach was well-suited to deal with the imbalanced outcome. The supplementary gain curve shows the model had high discriminatory power for all classes (the closer the black line is to the upper boundary of the gray triangle, the better the model) despite the class imbalance. There was a large overlap (i.e. 66.7%) in the variables contributing most to both prognostic models, but there were also some differences, mainly concerning prescribed pharmacotherapy, injury and medical treatment characteristics. Although univariate, the descriptive statistics of the most relevant predictors in the multinominal trajectory model indicate that these predictors differentially distinguish between trajectories. This is particularly important as it is currently impossible to differentiate between resilient versus delayed and between non-remitting versus recovery trajectories based on initial symptom severity, stressing the added value of the applied multi-nominal classification approach for early risk classification for PTSD and obtaining potential correlates of PTSD pathogenesis.

In both prognostic models previously reported group-level biomedical predictors of subsequent PTSD were amongst the most strongly contributing variables, including acute post-trauma SNS activity (Morris et al., 2016) and cortisol levels (Morris et al., 2016; Mouthaan et al., 2014a; Van Zuiden et al., 2012) and early pharmacotherapy with opiate analgesics (Mouthaan et al., 2015). Similarly, we replicated well-documented effects that age, prior traumatic events and perceived impact of these events (Brewin et al., 2000; Shalev et al., 2017), perceived life threat (Holbrook et al., 2001) and amnesia (Flesher et al., 2001) related to the traumatic event are associated with PTSD development.

Moreover, we also identified several new biomedical predictors of relevance for the prognostic models. To our best knowledge, we are the first to investigate the prognostic value of markers of the HPT-axis for PTSD development. The acute HPT-axis stress response is biphasic, with an initial brief increased functioning reverting into decreased functioning (Nadolnik, 2011), a process modulated amongst others by inhibitory effects of glucocorticoids. PTSD, especially in women, appears to be associated with increased thyroid function, (e.g. (Olff et al., 2006). Interestingly, all 3 investigated markers (TSH, FT4 and T3), were among the 15 most relevant predictors in the multinominal trajectory model, while TSH and FT4 were also among the 15 most relevant predictors in the end-point diagnostic model. The observed directionality aligns with the cross-sectional findings in PTSD patients, and the univariate descriptives for the multinominal model indicate similar associations for both the non-remitting and delayed courses. Although our observation needs replication, it suggests that altered HPT-axis function precedes PTSD development and may be involved in its pathogenesis. It was previously suggested that together with high SNS activity, high HPT-axis activity could contribute to PTSD's hyperarousal symptoms (Olff et al., 2006). This is of specific interest as two recent network analyses showed acute hyperarousal symptoms are central in the occurrence of PTSD symptoms from other clusters in the first days to weeks post-trauma (Bryant et al., 2017; Greene et al., 2018).

Our prognostic models also included several new categories of pharmacotherapy prescribed within the acute post-trauma period, on top of previously investigated effects of beta-adrenergic receptor blockers, opiates, systemic corticosteroids and benzodiazepines (Frijling et al., 2019). In the PTSD end-point model opiate analgesics, non-opiate analgesics and anesthetics were relevant pharmacotherapy categories for subsequent prognosis, providing additional support that adequate pain control is important to prevent adverse mental health outcome of injury (Norman et al., 2008). In the trajectory model antibiotics constituted the most relevant category, with more prescribed doses being especially associated with the delayed trajectory. This observation is noteworthy given the fact that the use of antibiotics affects the gut-microbiome and the accumulating evidence for associations between the gut-microbiome and psychiatric disorders, including PTSD (Hemmings et al., 2017). However prospective studies investigating whether the gut-microbiome is also associated in PTSD's development are not yet available, although there is increasing evidence for the involvement of inflammatory dysregulation in PTSD pathogenesis (Olff and van Zuiden, 2017). Intriguingly, there is preclinical evidence that administration of the antibiotic minocycline early post-trauma actually prevents PTSD-like symptom development in rodents (Wang et al., 2018). This finding is seemingly in contrast to our observation of higher doses of antibiotics being associated with increased risk for a delayed PTSD trajectory. However, the follow-up period of this preclinical study was only 3 weeks after the experimental trauma. Thus, it remains unknown what the more long-term effect of this preventive intervention would have been.

An alternative explanation may be that these observations reflect an unmeasured aspect of clinical decision making related to the severity of the sustained injuries, as higher physician-rated Injury Severity Score was significantly positively associated with a higher number of prescribed doses within the first 48 h post trauma for these medication categories (see Table S4).

Time of ED admission, a proxy variable for time of trauma exposure, was the second most important variable in the prognostic model for endpoint PTSD status and also was among the most important variables contributing to trajectory prognosis. A previous ML study forecasting remittance of initial high PTSD symptoms also revealed time of ED admittance to be relevant, although it could not be inferred which specific parts of the day were associated with non-remittance (Galatzer-Levy et al., 2014; Schultebraucks and Galatzer-Levy, 2019). From the SHAP analyses we can interpret that admittance at night was related to highest PTSD risk, followed by early morning, with lower risk when admitted during day or evening.

Notably, this association was observed while the model included other variables that may be related with trauma exposure during night, such as trauma type and cortisol levels upon ED admittance, implying other involved mechanisms. It is conceivable that participants that were ED admitted at night included shift-or night-time workers. Such work patterns are associated with chronic circadian misalignment and sleep disturbances (Rajaratnam et al., 2013), including altered endocrine stress reactivity (Koch et al., 2017). Also in the absence of non-traditional work patterns, nighttime trauma exposure may be specifically associated with acutely disturbed circadian rhythmicity and sleep. Circadian dysregulation is increasingly recognized as biological correlate of PTSD (Dayan et al., 2016), and genetic variation in circadian rhythm are related to PTSD risk (Linnstaedt et al., 2018). Additionally, pre-trauma blunted ultradian corticosterone patterns were found to be a vulnerability factor for PTSD-like behavior in rodents (Danan et al., 2018). Furthermore, chronic pre-trauma and acute post-trauma sleep disturbances have repeatedly been associated with PTSD vulnerability (Koffel et al., 2016). Our finding thus emphasizes the importance of more future research on the role of circadian and sleep dysregulation in the pathogenesis of PTSD (Morris et al., 2016; Van Zuiden et al., 2012).

We applied explainable ML to interpret the derived prognostic model for PTSD risk classification, in the form of SHAP values ranking the predictive variables for importance regarding their contributions to the prognostic model that prospectively distinguished ED patients likely to fulfil PTSD's diagnostic criteria at 12 months post-trauma from those who did not and to investigate their multivariate associations with PTSD outcome. Importantly, for all continuous predictors the observed classification decision rules for predicting end-point PTSD diagnostic status were non-linear across the range of predictor values, which has not been detected by previous group-level studies as they applied statistical methods testing linear associations. Relatedly, these differences in methodological approach can likely explain why some observed associations between predictors and long-term PTSD outcome, e.g. systolic blood pressure within ED, were in the opposite direction as previously observed (Morris et al., 2016). Additionally, this may also explain why acute DHEAS and time of ED admittance were of relevance for long-term PTSD risk classification in the current study but not significantly associated with long-term PTSD symptom severity in previous analyses within this cohort (Mouthaan et al., 2014a).

Thus, in addition to performing the first multinominal prognostic model and identifying several new predictors, we also extend previous findings by elaborating on the complex interactions and associations between several previously described predictors and dichotomous PTSD outcome, based on the gold standard clinical diagnostic interview for PTSD (CAPS). Moreover, the observed two-way interactions between the most contributing variables on the end-point classification were also non-linear. Additionally, our results indicate that the contributions of the biomedical information to the prediction of end-point classification PTSD, such as the contribution of time of ED admittance, TSH and cortisol, were dependent on prior trauma history. This interaction between prior trauma history and features such as TSH and cortisol adds to previous findings on the moderating effect of trauma history on the prognostic value of acute endocrine responses to trauma (Galatzer-Levy et al., 2017). Thus, together our findings also provide directions for further research to elucidate mechanisms contributing to PTSD pathogenesis and thus to increase our understanding of individual differences in vulnerability for PTSD upon traumatic events.

This naturalistic, prospective longitudinal multicenter study has a high external validity due to the communal exposure to (suspected) serious injury leading to ED admission. Of our participants completing the final assessment at 12 months post-injury, 4.4% fulfilled diagnostic criteria for current PTSD. At first view, our sample has a low PTSD prevalence compared to similar injury or acute care cohorts from other countries (Qi et al., 2018; Shalev et al., 2019). Yet, results from a mega-analysis including over 2400 participants from ten acute care cohorts across six countries, including the cohort used in the current study, indicate that the individual cohorts likely represent different sampling subsets of an underlying acute care population (Shalev et al., 2019). Thus, our cohort can be assumed to be a representative sample of acute care patients requiring ED admission. The majority of our participants were admitted to the ED following road traffic accidents. In the WHO World Mental Health Surveys performed across high-, middle-, and low-income countries, it was found that motor vehicle collisions perceived as life-threatening constituted the fourth most common type of traumatic event reported across countries. The prevalence of current PTSD associated with life-threatening motor vehicle collisions was 2.5% and did not vary significantly across countries. Although this prevalence may seem relatively low, given the fact that worldwide road traffic accidents result in more than 20 million non-fatal injuries annually (Ameratunga et al., 2006), PTSD incidence related to road traffic accidents nevertheless constitutes an important global public health problem.

We applied repeated in-depth phenotyping to our cohort. The included physiological and endocrine data were collected immediately upon ED admittance and thus reflect acute traumatic stress responses. Thus far, only few prospective studies have collected such acute data. These data were combined with data from other domains previously found to be relevant for predicting PTSD at group-level (Heim et al., 2018; Morris et al., 2016; Ressler, 2018; Shalev et al., 2017; Van Zuiden et al., 2012) and in previous ML studies predominantly predicting chronic PTSD course as outcome of interest (Schultebraucks and Galatzer-Levy, 2019). Thus, although ML methods enabled complex data-driven multivariate predictions, the candidate predictors were still informed by existing literature and thus hypothesis-driven. This improves the clinical validity of our model, especially as we also used explainable ML to facilitate their interpretation and critical appraisal. It is important to note that the feature importance ranking cannot be interpreted causally, and the prediction is always a combination of all variables together.

Although the sample size limits the generalizability of the results to the broad ED population beyond our defined inclusion and exclusion criteria, we applied established measures to guard against overfitting by keeping the discovery dataset and test dataset strictly apart and by applying cross-validation during model development on the discovery dataset. Moreover, a subgroup of our participants also participated in an embedded RCT investigating the efficacy of a preventive intervention, consisting of brief self-guided internet intervention based on cognitive behavioral therapy techniques (Mouthaan et al., 2013). RCT participation status and condition were not significantly associated with end-point PTSD diagnostic status nor with self-reported PTSD symptom course within the observed trajectories. Moreover, sensitivity analyses revealed excluding the participants randomized into the intervention condition did not result in altered performance of the prognostic model for end-point PTSD. Therefore, we consider the impact of this embedded RCT on the current results to be limited.

The prognostic models should be replicated in an independent ED sample of acute injury patients before considering potential implementation in the future. As in the current study, patients with immediate pre-trauma severe mental health problems and moderate to severe traumatic brain injuries were excluded, it remains to be investigated whether the derived prognostic models generalize to a broader ED sample. Additionally, it would be of interest to investigate whether prognostic models using acute biomedical data upon occurrence of other events requiring acute medical care, such as large scale crises (e.g. infectious disease outbreaks),cardiovascular events (Musey et al., 2019; Schultebraucks et al., 2020b) are equally informative regarding early risk classification for PTSD. Also, it remains to be investigated whether the prognostic models would generalize to combat-sustained injuries.

The identified prognostic models accurately forecasted PTSD symptom severity course and end-point PTSD diagnostic status by only using biomedical data collected immediately upon arrival in the ED and within the first 48 h after admission. All information included in the models is easily collectable and either is or could be routinely included in electronic medical records upon ED admittance. Therefore, these prognostic models could have high clinical utility. If replicated, our results have important public health implications and inform new strategies to optimize efficient targeted allocation of preventive interventions early post-injury. In this early time window between injury and manifestation of full-blown PTSD, several critical pathogenetic processes take place and preventive interventions may be most effective (Shalev et al., 2019). The early contact with the health care system after acute injury or other acute medical events provides a precious opportunity for such preventive interventions. It was repeatedly observed that especially those at high risk for long-term PTSD benefit from preventive interventions (Shalev and Barbano, 2019). Our prognostic models for PTSD risk classification would allow for time-efficient support of clinical decision-making by indicating which individuals could benefit most from offering preventive interventions, such as easily accessible, low-threshold internet-based interventions (Mouthaan et al., 2013). The applied explainable ML method additionally allows clinicians to interpret and critically appraise the obtained individual prognoses in light of their expertise. Accurate targeted allocation of preventive interventions towards those at highest risk could prevent long-term adverse outcome, improve overall functioning and reduce mental health care use associated with PTSD.

In addition, this study also shows that computational multivariate modeling using ML can contribute to increased understanding of biological pathways underlying individual differences in vulnerability for long-term PTSD. Our findings provide directions for further research to elucidate mechanisms by which the identified predictors may contribute to PTSD pathogenesis and if therapeutic strategies modifying these predictors may be effective targeted preventive interventions.

## Role of the funding source

The TraumaTIPS study was supported by ZonMw, the Netherlands Organization for Health Research and Development (grant #62300038), the Hague, the Netherlands, and by Stichting Achmea Slachtoffer en Samenleving (SASS), Aid to Victims, Zeist, the Netherlands. The current project was additionally supported by an Amsterdam Public Health Research Institute Alliance Grant, Amsterdam, the Netherlands, within the Mental Health Program. Katharina Schultebraucks was supported by the German Research Foundation (SCHU 3259/1-1). Mirjam van Zuiden was supported by a Veni grant from ZonMw, the Netherlands organization for Health Research and Development (#91617037), the Hague, the Netherlands.. The funders were not involved in the collection, analysis and interpretation of data; in the writing of the report; and in the decision to submit the article for publication.

## CRediT authorship contribution statement

**Katharina Schultebraucks:** Methodology, Software, Formal analysis, Visualization, Writing - original draft. **Marit Sijbrandij:** Conceptualization, Investigation, Funding acquisition, Writing - review & editing. **Isaac Galatzer-Levy:** Methodology, Writing - review & editing. **Joanne Mouthaan:** Investigation, Project administration, Writing - review & editing. **Miranda Olff:** Conceptualization, Data curation, Investigation, Supervision, Writing - review & editing. **Mirjam van Zuiden:** Conceptualization, Data curation, Formal analysis, Funding acquisition, Methodology, Writing - original draft, Supervision, Funding acquisition.

## Declaration of competing interest

The authors declare no conflict of interest.
