# Supplementary material for: Forecasting individual risk for long-term Posttraumatic Stress Disorder in emergency medical settings using biomedical data: A machine learning multicenter cohort study
Source: Neurobiol Stress. 2021 Jan 18;14:100297. doi: 10.1016/j.ynstr.2021.100297 (PMC7843920; doi:10.1016/j.ynstr.2021.100297)
Supplement: Multimedia component 1 [file mmc1.docx]

**Supplementary Methods**

**Figure S1.** Schematic overview of study design

**
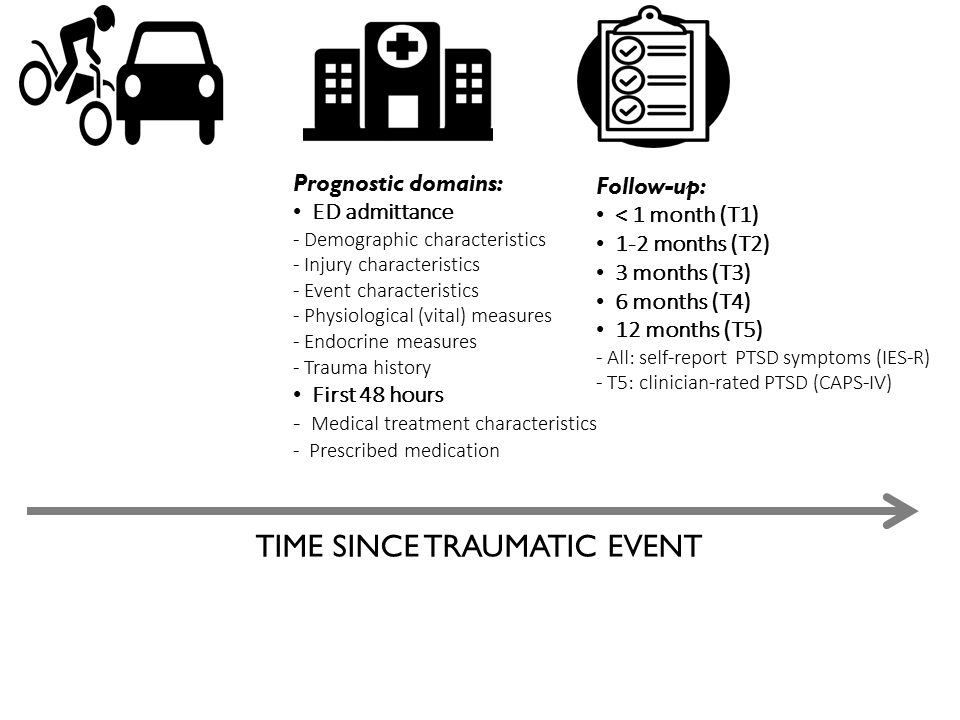
**

**Supplementary Results**

**Table S1.** Latent Growth Mixture Model fit indices for self-reported PTSD symptom trajectories

|  |  | **Number of classes** | | | | | | | | | |
| --- | --- | --- | --- | --- | --- | --- | --- | --- | --- | --- | --- |
| **Fit indices** |  | 1 | 2 | 3 | 4 | 5 | 1 | 2 | 3 | 4 | 5 |
|  |  | **Models with linear slopes** | | | | | **Models with linear and quadratic slopes** | | | | |
|  | AIC | 9588 | 9337 | 9241 | 9175 | 9128 | 9538 | 9291 | 9192 | 9129 | 9050 |
|  | BIC | 9625 | 9386 | 9302 | 9247 | 9213 | 9578 | 9347 | 9264 | 9218 | 9155 |
|  | Sample Size- adjusted BIC | 9596 | 9348 | 9254 | 9190 | 9146 | 9546 | 9303 | 9207 | 9148 | 9073 |
|  | Entropy | – | 0.964 | 0.894 | 0.913 | 0.910 | – | . 964 | .901 | .900 | .901 |
|  | VLRT | – | <.001 | <.0012 | <.001 |  | – | <.001 | <.001 | <.001 |  |

All models contained freely estimated intercept, slope and quadratic slope, with quadratic slope variance fixed to zero.

**Table S2.** Differences in clinical outcomes as assessed 12 months post-trauma (T5) between the identified latent PTSD symptom trajectories.

|  | Class 1:  resilient | Class 2: recovery | Class 3: delayed | Class 4: chronic | Omnibus Test | Post hoc:  4 vs 1 | Post hoc:  4 vs 2 | Post hoc:  4 vs 3 | Post hoc:  3 vs 1 | Post hoc:  3 vs 2 | Post hoc:  2 vs 1 |
| --- | --- | --- | --- | --- | --- | --- | --- | --- | --- | --- | --- |
| PTSD diagnosis  *no*  *yes* | 222 (98.7%)  3 (1.3%) | 23 (100%)  0 (0%) | 10 (71.4%)  4 (28.6%) | 6 (54.5%)  5 (45.5%) | F(3): 35.203, p<.001 | p<.001* | p=.002* | p=.434 | p<.001* | p=.015* | p=1.000 |
| CAPS total score | 9.48 (9.19) | 18.78 (9.71) | 30.31 (15.32) | 43.82 (33.89) | F(3,269): 45.848, p<.001 | p<.001* | p=.003* | p=.004* | p<.001* | p=.003* | p<.001* |
| Quality Of Life Psychological health Domain | 15.84 (2.35) | 14.73 (2.50) | 12.67 (2.00) | 11.60 (3.39) | F(3,237): 16.728, p<.001 | p<.001* | p=.001* | p=.291 | p<.001* | P=.015* | p=.046 |
| Quality Of Life Social Relationships Domain | 15.95 (2.62) | 14.41 (3.34) | 13.33 (2.49) | 11.60 (3.88) | F(3,237):  12.312, p<.001 | p<.001* | p=.008* | p=.134 | p=.001* | P=.226 | p=.015* |

Obtained scores are presented as mean (SD) for continuous measures and N (%) for categorical measures. Upon a significant omnibus test (i.e. ANOVA or Bootstrapped Fishers exact test), we contrasted all classes against each other and corrected for multiple testing using the Holm-Bonferroni procedure, meaning that for each outcome the p-values were ranked from lowest to highest, and compared to increasing alpha levels of significance (i.e. p<0.0021, 0.0042, 0.0084 , 0.0167; 0.025, 0.05 respectively). Posthoc tests that remained significant upon corrected are designated with *.

**Figure S2.** Cumulative gain curve for the four PTSD symptom trajectories through 12 months after Emergency Department admission.

**

The cumulative gain curve (Engelmann et al., 2003; Kuhn and Johnson, 2013) also known as lift plot,(Ling and Li, 1998) shows the gains associated with using the model, comparing its performance with how successful we would be without the added value offered by the model. The cumulative gain (also known as accuracy ratio) is calculated as the area under the black curve (SFigure 2), but above the 45 degree line, divided by the area of the gray triangle.(Engelmann et al., 2003; Kuhn and Johnson, 2013) We order all participants according to the predicted probability of the model. On the left-hand side of the x-axis are the highest predicted probabilities of the respective class according to the model while the lowest probabilities are on the right-hand side. The hypotenuse of the gray triangle (the 45° reference line) represents the random model (a non-informative model), while the catheti represent the perfect model. The x-axis represents the rate of participants tested as percentage and the y-axis represents the rate of positive events (the respective class) detected. As the shape of the gray triangle depends on the distribution of positive events in the test set, this metric is useful for imbalanced samples. It indicates the direct gain of using the model compared to not using the model (45-degree line). The prediction of all four classes have a marked positive gain, which indicates that the models are useful. The macro weighted Area Under the Cumulative Gain Curve is 0.85.

**Figure S3.** The cumulative gain curve for the prediction of CAPS-IV PTSD caseness at 12 months after Emergency Department admission. The Area Under the Cumulative Gain Curve is 0.79.


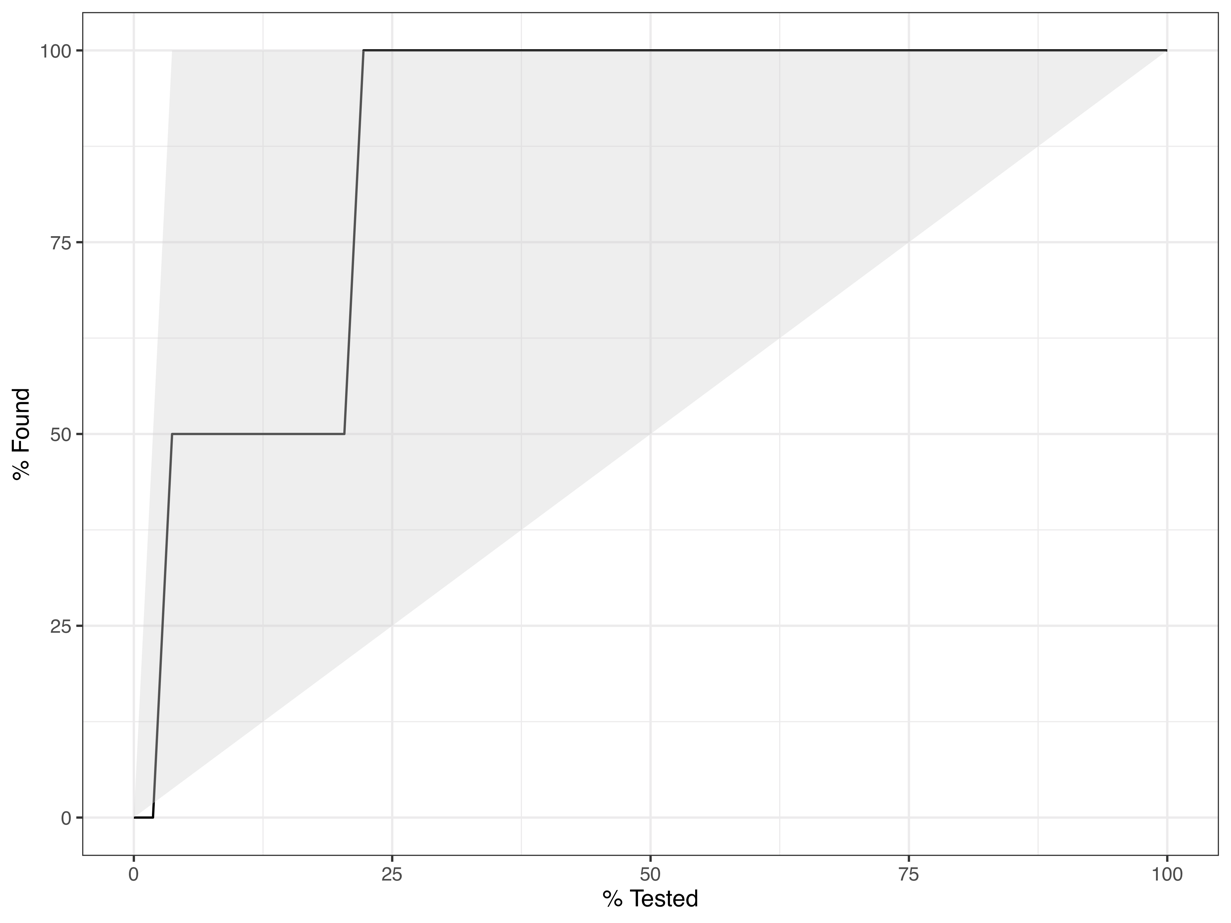


**Figure S4.** Display of the top 15 variables using variable importance ranking to predict PTSD diagnostic status using CAPS-IV at 12 months after Emergency Department admission for the hold-out set using SHapley Additive exPlanations (SHAP)*.* The plot represents the mean absolute SHAP value per feature, with larger SHAP values representing a higher importance to differentiate between PTSD and non-PTSD diagnosis with CAPS-IV at 12 months.

**Figure S5.** Display of the top 15 feature using variable importance ranking to predict PTSD symptom trajectory membership over 12 months after Emergency department admission for the hold-out set using the model specific variable importance ranking implemented in the R package caret (Kuhn, 2008). The plot represents the variable importance per feature, with larger values representing a higher importance to differentiate between trajectory membership.

**Table S3.** Descriptives for the 15 most important variables contributing to the model predicting PTSD diagnostic status at 12 months after Emergency Department admission. Participants were separated into groups based on their end-point CAPS-IV PTSD diagnostic status and descriptives for the variables per group were derived from the raw data. Descriptives are presented as mean (SD) for continuous variables and percentage for dichotomous variables.

|  | **End-point PTSD (n=12)** | **No end-point PTSD (n=261)** |
| --- | --- | --- |
| TSH (mE/L) | 1.26 (0.54) | 5.85 (5.10) |
| Time admitted to Emergency Department | 11:59 am (6:15) | 2:09 pm (5:10) |
| Cortisol (nmol/L) | 653.88 (144.16) | 761.04 (271.97) |
| Perceived threat of own life (yes) | 40% | 19.6% |
| DHEAS (nmol/L) | 5.38 (1.72) | 4.31 (3.10) |
| Systolic blood pressure | 139.00 (15.38) | 141.42 (25.49) |
| Total impact of prior traumatic events | 7.00 (5.98) | 4.86 (5.10) |
| Free T4 (pmol/L) | 15.19 (2.88) | 11.87 (5.92) |
| Age | 40.28 (8.45) | 46.18 (16.07) |
| Non-opiate anesthetics (doses) | 0.63 (0.74) | 1.27 (1.37) |
| Amnesia (self-reported, yes) | 50.0% | 45.3% |
| Non-opiate analgesics (doses) | 0.63 (0.74) | 1.43 (2.28) |
| Opiate analgesics (doses) | 0.38 (0.52) | 0.59 (0.90) |
| Prior traumatic events (number of types) | 4.75 (2.93) | 2.64 (2.24) |
| Pulse | 82.13 (21.94) | 82.40 (17.65) |

**Figure S6.** Partial dependence plot (Friedman, 2001) of probability for end-point PTSD (red color) and non-PTSD (blue color), and interaction between the most important variables contributing to the model predicting diagnostic status at 12 months after Emergency Department admission (i.e, TSH, Time admitted to Emergency Department (Time.EDadmit), cortisol, perceived threat of own life (thought.would.die.yes) and number of prior traumatic events. For instance, the likelihood of PTSD is higher for patients with lower cortisol levels and an earlier time of admittance (red color), whereas higher cortisol and later time of admission is related to a higher likelihood of non-PTSD (blue color).

**Table S4.** Descriptives for the 15 most important variables contributing to the model predicting PTSD symptom trajectory membership over 12 months after Emergency Department admission. Participants were separated into groups based on their assigned latent trajectory membership in the latent growth mixture model and descriptives for the variables per group were derived from the raw data. Descriptives are presented as mean (SD) for continuous variables and percentage for dichotomous variables.

|  | **Resilient (n=339)** | **Recovery (n=37)** | **Delayed onset (n=20)** | **Non-remitting (n=21)** |
| --- | --- | --- | --- | --- |
| Antibiotics use (doses) | 0.28 (0.89) | 0.32 (0.75) | 0.45 (0.69) | 0.35 (0.86) |
| Glascow Coma Scale | 14.39 (2.16) | 14.40 (2.40) | 13.92 (3.33) | 14.63 (0.81) |
| Cortisol (nmol/L) | 780.93 (260.61) | 674.47 (188.76) | 685.70 (239.88) | 694.27 (214.85) |
| Age | 45.98 (15.91) | 43.87 (15.16) | 52.78 (12.08) | 45.39 (19.02) |
| DHEAS (nmol/L) | 4.62 (3.28) | 4.10 (2.25) | 4.20 (2.80) | 4.82 (2.48) |
| Pulse | 82.37 (17.24) | 74.06 (14.41) | 91.88 (17.84) | 74.29 (13.46) |
| Free T3 (pmol/L) | 1.77 (0.61) | 1.84 (0.53) | 1.54 (0.34) | 1.65 (0.17) |
| TSH (mE/L) | 5.80 (5.95) | 5.29 (5.63) | 3.92 (5.29) | 2.67 (3.43) |
| Free T4 (pmol/L) | 11.98 (5.74) | 11.51 (5.88) | 16.12 (4.05) | 13.71 (5.06) |
| Prior traumatic events (number) | 2.69 (2.12) | 3.32 (3.06) | 3.00 (1.73) | 3.58 (3.01) |
| Time admitted to ED | 1:41 pm (5:34) | 1:57 pm (5:41) | 1:30 pm (5:13) | 12:00 pm (6:29) |
| ICU admission (yes) | 9.2% | 11.1% | 16.7% | 15.8% |
| Head injury sustained (yes) | 53.3% | 62.2% | 57.9% | 68.4% |
| Systolic blood pressure | 143.60 (24.98) | 146.78 (29.74) | 138.89 (33.91) | 138.33 (16.15) |
| Opiate analgesics (doses) | 0.58 (0.94) | 0.84 (0.90) | 0.27 (0.47) | 0.52 (0.80) |

**Table S5.** Correlations between Injury Severity Score and early pharmacotherapy categories included in the prognostic model, quantified as the number of doses prescribed by the hospital within 48 hours post-injury.

|  | ***Spearman Rho correlation coefficient with Injury Severity Score*** |
| --- | --- |
| *Opiate analgesics (doses)* | .427*** |
| *Non-opiate analgesics (doses)* | .415*** |
| *Non-opiate anesthetics (doses)* | .415*** |
| *Anticoagulators (doses)* | .477*** |
| *Antibiotics (doses)* | .243*** |
| *Benzodiazepines (doses)* | .106 |

*** p<.001.

**Secondary analyses regarding impact of embedded RCT**

*Participant characteristics*

There were no differences in socio-demographic and injury characteristics between RCT participants in the control and intervention condition and non-RCT participants. Regarding trauma characteristics, RCT-intervention participants were less likely to report perceived life threat during their traumatic event prior to randomization (11.1% vs 27.5% of RCT-controls and 21.3% of non-RCT participants, X^2^(2) = 7.411, p=.025). RCT-participants in the control condition were more commonly confronted with injured individuals (21.3%) during the traumatic events than RCT-intervention participants (9.8%) and non-RCT participants (10.4%) (X^2^(2) = 7.094, p=.029).

*PTSD symptom trajectories*

As adding the effects of two dummies for RCT condition on intercept, linear slope and quadratic slope to the LGMM model resulted in model nonidentification, we continued with adding the effects to the intercept and linear slope only. This did not result in improved model fit (AIC: 9146, BIC: 9251, Sample-size adjusted BIC: 9169) compared to the basic model without these effects (see supplementary table 1). Compared to the reference group of non-RCT participants, participants in the intervention and control conditions did not have significantly different intercepts and linear slope across trajectories (intercept on control condition: Estimate (S.E.) 0.879 (1.831), Z: 0.480, p=.631; intercept on intervention condition: Estimate (S.E.) -2.168 (1.178), Z: -1.841, p=.066; slope on control condition: Estimate (S.E.) -0.004 (0.004), Z: -1.175, p=.6240; slope on intervention condition: Estimate (S.E.) 0.005 (0.003), Z: -1.780, p=.075).

*Sensitivity analysis prognostic models*

**Figure S7.** Receiver-Operating Characteristic Curve for predicting PTSD diagnosis at 12 months after ED admission for a sensitivity analysis containing only participants not participating in the RCT or randomized to the control group within the RCT (AUC = .88 (95% CI: 0.75-1.00, sensitivity = 1.00, specificity. = 0.83, precision = 0.96).

**References**

Engelmann, B., Hayden, E., Tasche, D., 2003. Measuring the discriminative power of rating systems. Discussion paper, Series 2: Banking and Financial Supervision.

Friedman, J.H., 2001. Greedy function approximation: a gradient boosting machine. Annals of statistics, 1189-1232.

Kuhn, M., 2008. Building Predictive Models in R Using the caret Package. 2008 28, 26.

Kuhn, M., Johnson, K., 2013. Applied predictive modeling. Springer.

Ling, C.X., Li, C., 1998. Data mining for direct marketing: Problems and solutions. Kdd, pp. 73-79.
